# Oncolytic targeting of androgen-sensitive prostate tumor by the respiratory syncytial virus (RSV): consequences of deficient interferon-dependent antiviral defense

**DOI:** 10.1186/1471-2407-11-43

**Published:** 2011-01-28

**Authors:** Ibtissam Echchgadda, Te-Hung Chang, Ahmed Sabbah, Imad Bakri, Yuji Ikeno, Gene B Hubbard, Bandana Chatterjee, Santanu Bose

**Affiliations:** 1 Department of Microbiology and Immunology, The University of Texas Health Science Center at San Antonio, 7703 Floyd Curl Drive, MC-7758, San Antonio, TX 78229, USA; 2 Department of Molecular Medicine/Institute of Biotechnology, the University of Texas Health Science Center at San Antonio, 15355 Lambda Drive, San Antonio, TX 78245, USA; 3 Department of Pathology, The University of Texas Health Science Center at San Antonio, 7703 Floyd Curl Drive, San Antonio, TX 78229, USA; 4 South Texas Veterans Health Care System, 400 Merton Minter, San Antonio, Texas 78229, USA

## Abstract

**Background:**

Oncolytic virotherapy for cancer treatment utilizes viruses for selective infection and death of cancer cells without any adverse effect on normal cells. We previously reported that the human respiratory syncytial virus (RSV) is a novel oncolytic virus against androgen-independent PC-3 human prostate cancer cells. The present study extends the result to androgen-dependent prostate cancer, and explores the underlying mechanism that triggers RSV-induced oncolysis of prostate cancer cells.

**Methods:**

The oncolytic effect of RSV on androgen-sensitive LNCaP human prostate cancer cells and on androgen-independent RM1 murine prostate cancer cells was studied *in vitro *in culture and *in vivo *in a xenograft or allograft tumor model. *In vitro*, cell viability, infectivity and apoptosis were monitored by MTT assay, viral plaque assay and annexin V staining, respectively. *In vivo *studies involved virus administration to prostate tumors grown in immune compromised nude mice and in syngeneic immune competent C57BL/6J mice. Anti-tumorogenic oncolytic activity was monitored by measuring tumor volume, imaging bioluminescent tumors in live animals and performing histopathological analysis and TUNEL assay with tumors

**Results:**

We show that RSV imposes a potent oncolytic effect on LNCaP prostate cancer cells. RSV infectivity was markedly higher in LNCaP cells compared to the non-tumorigenic RWPE-1 human prostate cells. The enhanced viral burden led to LNCaP cell apoptosis and growth inhibition of LNCaP xenograft tumors in nude mice. A functional host immune response did not interfere with RSV-induced oncolysis, since growth of xenograft tumors in syngeneic C57BL/6J mice from murine RM1 cells was inhibited upon RSV administration. LNCaP cells failed to activate the type-I interferon (IFNα/β)-induced transcription factor STAT-1, which is required for antiviral gene expression, although these cells could produce IFN in response to RSV infection. The essential role of IFN in restricting infection was further borne out by our finding that neutralizing IFN activity resulted in enhanced RSV infection in non-tumorigenic RWPE-1 prostate cells.

**Conclusions:**

We demonstrated that RSV is potentially a useful therapeutic tool in the treatment of androgen-sensitive and androgen-independent prostate cancer. Moreover, impaired IFN-mediated antiviral response is the likely cause of higher viral burden and resulting oncolysis of androgen-sensitive prostate cancer cells.

## Background

Oncolytic virotherapy, which takes advantage of selective viral infection and apoptosis in cancer cells due to robust viral replication, is emerging as an important alternative to conventional cancer treatment modalities [1-4]. Evidence indicates that concurrent use of a repertoire of different oncolytic viruses (with different modes of action) may produce more efficacious therapeutic response. Human respiratory syncytial virus (RSV) is a respiratory tract-specific enveloped non-segmented negative sense single stranded RNA (NNS) virus of the paramyxovirus family [5,6]. We have recently identified RSV as an oncolytic virus by demonstrating that RSV can cause apoptosis of PC-3 human prostate cancer cells in culture and in a xenograft tumor environment as a consequence of excessive viral replication in PC-3 cells that led to apoptotic cell death [7]. Specificity of the virus-induced oncolysis of cancer cells was evident from the lack of significant viral burden and apoptosis of non-tumorigenic human prostate epithelial cells, such as RWPE-1.

Metastatic prostate cancer is a leading cause of cancer deaths in men in the United States. The steroid hormone androgen is a potent mitogen for normal and cancerous prostate epithelial cells. The cognate androgen receptor (AR) mediates nuclear responses to androgen signaling [8,9]. Although initially androgen-sensitive, metastatic prostate cancer evolves to a state of androgen independence for growth and proliferation, despite continued expression of AR at all stages of the disease. AR was shown to activate the transcriptional program of a distinct set of gene networks, including many genes involved in cell cycle regulation, during progression of the cancer cells to androgen independence [9]. As noted above, RSV can induce oncolysis of androgen-independent PC-3 prostate cancer cells and RSV caused regression of PC-3 cell derived xenograft tumors in immune-deficient nude mice [7]. Extending this study, we examined susceptibility of the androgen-sensitive LNCaP human prostate cancer cells and LNCaP xenograft tumors to RSV-induced oncolysis, and the impact of host immune-competence on the oncolytic activity of RSV. Innate immunity is the first line of antiviral defense for restricting virus growth and spread. Since both NF-κB and type-I interferon (IFNα/β)-mediated JAK/STAT signaling [10] is required for innate antiviral response; we also examined the activation status of NF-κB and IFN-induced JAK-STAT pathways in RSV-infected prostate cancer cells.

Herein we report that RSV is potently oncolytic against androgen-sensitive LNCaP human prostate cancer cells *in vitro *and *in vivo*, and aberrant IFN-regulated signaling accounts for LNCaP cell susceptibility to RSV. While RWPE-1 non-tumorigenic prostate cells were protected against RSV infection by activation of JAK-STAT and NF-κB signaling, a lack of sustained NF-κB activation was associated with the susceptibility of PC-3 androgen-independent prostate cancer cells to RSV-induced oncolysis, although IFN-mediated signaling was functional in these cells.

## Methods

### Virus, Cells, Culture Conditions, Chemicals

RSV (A2 strain, ATCC, Manassas, VA) was propagated in CV-1 cells. Viral titer was monitored by plaque assay [7]. LNCaP, PC-3 and RWPE-1 (source: ATCC) were cultured as per the supplier's instructions. RM1 murine prostate cancer cells were cultured in DMEM [11,12]. RM1 cells (a gift from Dr. Timothy Thompson, Baylor College of Medicine, TX) originated from stable expression of Ha-Ras and c-Myc oncogenes in mouse prostate epithelial cells [11,12]. RM1 cells express androgen receptor but they are androgen-independent. Interferon-α (IFN-α) and Tumor necrosis factor-α (TNF-α) were purchased from R&D Systems (Minneapolis, MN).

### RSV infection, interferon (IFN) treatment, ELISA for IFN-β, IFN-γ and IL-10

RSV (1 MOI) was added to cells for adsorption at 37°C for 1.5 h. After washing, infection continued for additional 0 h-48 h. At various time points post-infection, virus yields in culture supernatants were assayed by plaque assay of the monolayer of CV-1 cells (African green monkey kidney cells) covered with a nutrient medium in methyl-cellulose [7]. Crystal violet staining of living cells allowed clear visualization of the plaques. Cell morphology was visualized microscopically. In some experiments, cells were pre-treated with 1000 units/ml IFN-α for 16 h, followed by infection with RSV for 24 h in the presence of IFN. Medium supernatants were used to measure viral titer (plaque assay).

Medium supernatants from RSV-infected cells at 16 h post-infection were analyzed for IFN-β levels using a human IFN-β specific ELISA kit (PBL Interferon Source, NJ). In some experiments, spleen homogenate and tumor homogenate obtained from mice were analyzed for IFN-γ and IL-10 levels using FACS based cytokine bead array (BD Biosciences, San Jose, CA).

### Apoptosis, cell viability

Cells infected with RSV for 24 h-36 h, were examined for apoptosis by annexinV labeling, using annexin V/propidium iodide apoptosis detection kit (BioVision, CA) and for cell viability using MTT (3-(4,5-dimethylthiazol-2-yl)-2,5-diphenyltetrazolium bromide) assay [13].

### Xenograft prostate tumors in nude mice and allograft tumors in syngeneic C57BL/6J mice

4-6-week-old athymic nude male mice (Harlan Laboratories Inc.) were injected subcutaneously with 3 × 10^6 ^LNCaP cells at a site below the ear [7]. When tumors reached palpable size (sizes ranging from 100-300 mm^3 ^for individual mice), RSV (at 1 × 10^6 ^pfu per animal) or Opti-MEM (Medium, carrier control) was injected repeatedly into the tumor mass (intratumoral administration) at 2-day intervals over a two-week period. Tumor volumes were measured by a caliper (4/3 × 3.14 × r_1_^2 ^× r_2 _with r_1 _< r_2_) and normalized to the tumor volume for the corresponding mouse at day-1 when the first injection was administered. Tumor samples from euthanized mice were excised and processed for histology and for TUNEL assay to evaluate apoptosis.

For imaging of subcutaneous xenograft tumors, which were generated using luciferase-expressing LNCaP-Luc-2 cells, palpable tumors were injected with RSV (1 × 10^6 ^pfu per animal via the intratumoral (I.T.) route) or Opti-MEM (Medium, carrier control). At various days post-infection, luciferin was injected and real-time tumor bioluminescence (reflecting tumor growth) was monitored in live animals using the Xenogen IVIS imaging system (Caliper Life Sciences, Hopkinton, MA).

To analyze prostate tumors in syngeneic C57BL/6J mice, 4-6 week-old mice were injected subcutaneously with RM1 murine prostate cancer cells (1 × 10^6 ^cells) in the right dorsal flank. Tumors (tumor volumes ranging from 56-164 mm^3 ^for individual mice) were injected intra-tumorally with either RSV (at 1 × 10^6 ^pfu, each animal) or Opti-MEM. The tumor volume for each animal was normalized to the corresponding volume recorded just prior to the first injection.

Housing and all procedures involving animals were performed according to protocols approved by the Institutional Committee for Animal Care and Use of the University of Texas Health Science Center San Antonio.

### Electrophoretic gel mobility shift (EMSA)

RSV-infected (0-24 h) or mock-infected cells were processed for nuclear fraction enriched total cell extracts [14]. In some cases, cells were treated with IFN-α (1000 units/ml, 4 h) or TNF-α (30 ng/m, 2 h). Nuclear extracts were incubated with ^32^P-labeled oligonucleotide duplex for cis-elements of NF-κB (IL6 promoter) or STAT-1 (hSIE/m67, synthetic c-sis-inducible element). Protein-DNA complex was analyzed as before [15]. NFκB element: 5'-GGGAATTCCCCATCTACGCTA; STAT-1 element: 5'-GTCGACA-TTTCCCGTAAATC.

### Histopathology and TUNEL

Part of tumor tissues (excised immediately after euthanasia) was fixed in 10% neutral buffered formalin, embedded in paraffin, sectioned at 5 micron and analyzed for histology after staining with hematoxylin and eosin. TUNEL staining *in situ *was performed using DeadEnd Colorimetric kit (Promega, WI). For each tumor sample, pathologic changes were graded by a blinded board certified veterinary pathologist (GBH). Grading criteria were based on invasion of adjacent normal tissue, necrosis, mitotic figures per 20X high power field, edema, congestion, compression of the surrounding tissue, mineralization, inflammation (granulocytes, lymphocytes, mixed), hemorrhage, neovascularization and hemosiderin deposition.

### Statistics

Differential tumor growth was determined utilizing STATA Analysis and Statistical Software (College Station, Texas, USA). Results are mean values ± Standard Error (SE) of the mean. For each mouse, the tumor volume at every time point was normalized to its tumor volume at the start of RSV (or Medium) injection, which was set as 100%. Significance of the difference between Medium- and RSV-injected tumor growth trajectories was evaluated using linear mixed model that incorporated a random intercept for each mouse and used normalized mean tumor volume measurements as the dependent variable. Wald tests assessed statistical significance of differences between growth rates in two mouse groups.

## Results

### RSV-induced oncolysis of LNCaP and RM1 cells

**Figure 1:**
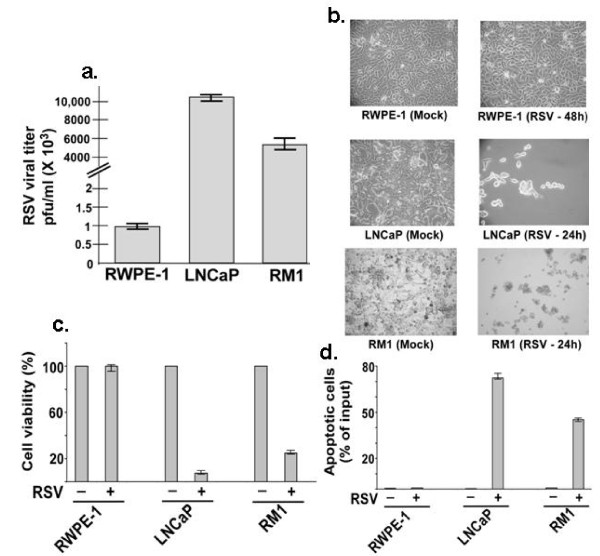
**Oncolytic activity of RSV**. **(a) **RSV infection of RWPE-1, LNCaP and RM1 cells were measured by plaque assay at 36 h post-infection. Plaque assay values expressed as pfu/ml represent mean ± standard deviation for three independent determinations. Standard deviations are shown by the error bars. **(b) **Morphology of mock-infected or RSV-infected cells at the indicated post-infection time periods. **(c) **MTT cell viability assay of cells infected with RSV for 36 h. MTT assay values are mean ± standard deviation of 6 wells and triplicate experiments. Uninfected (-) cells indicate 100% cell viability. **(d) **RSV mediated apoptosis at 24 h post-infection. % Apoptosis represents the % of cells that are undergoing apoptosis (i.e. positive for annexin V staining). The values represent mean ± SD for three determinations.

Figure 1 show that similar to PC-3 androgen-independent prostate cancer cells RSV induces apoptosis due to its oncolytic activity against androgen-sensitive LNCaP prostate cancer cells and RM1 murine prostate cancer cells. Apoptosis was minimal for RWPE-1 non-malignant prostate epithelial cells. Compared to RWPE-1, RSV titer was drastically enhanced in LNCaP and RM1 cells (Figure 1a). This led to increased cytopathicity (evident from cell rounding and loss of normal cellular morphology) (Figure 1b) and reduced cell viability (Figure 1c). Reduced cell viability was due to increased apoptosis of RSV infected LNCaP and RM1 cells (Figure 1d). Cancer cell-selective apoptosis relative to non-tumorigenic RWPE-1 prostate cells indicated that RSV imparts oncolytic activity on LNCaP and RM1 cells.

### RSV-induced oncolysis of androgen-dependent and androgen-independent prostate cancer cells in the absence and presence of androgen

**Figure 2:**
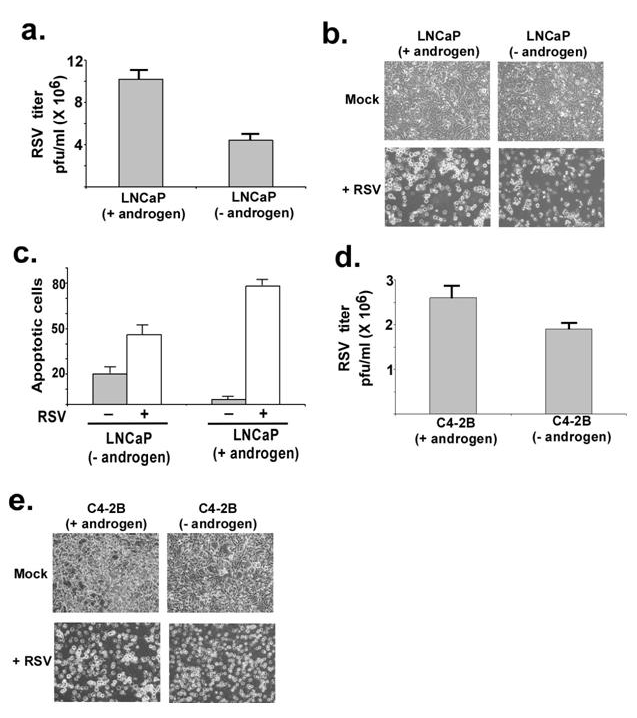
**Oncolytic activity of RSV against androgen-dependent and androgen-independent prostate cancer cells in the absence and presence of androgen**. **(a) **RSV infection of LNCaP cells (in the presence or absence of androgen) were measured by plaque assay as described in Figure. 1a. Standard deviations are shown by the error bars. **(b) **Morphology of mock-infected or RSV-infected LNCaP cells (in the presence or absence of androgen). **(c) **Apoptosis of mock-infected (- RSV) and RSV-infected (+ RSV) LNCaP cells (in the presence or absence of androgen). Apoptosis assay was performed as described in Figure 1 d. The figure shows the percentage of cells undergoing apoptosis. Standard deviations are shown by the error bars. **(d) **RSV infection of C4-2B cells (in the presence or absence of androgen) were measured by plaque assay as described in Figure. 1a. Standard deviations are shown by the error bars. **(e) **Morphology of mock-infected or RSV-infected C4-2B cells (in the presence or absence of androgen).

To assess the role of androgen in the RSV effect on LNCaP (androgen-dependent) and C4-2B (androgen-independent, derived from the parental LNCaP) cells, the cells were grown in androgen-depleted (charcoal stripped) media and then either ethanol (vehicle) or R1881a (1 nM) was added to the media for overnight before mock infection or infection with RSV was performed. RSV replicated efficiently in vehicle-treated and R1881 (1 nM)-treated LNCaP and C4-2B prostate cancer cells (Figure 2a, d). The RSV titer was higher in cells with androgen treatment compared to vehicle treatment. Cell morphology (Figure 2b, e) revealed that RSV caused oncolysis of LNCaP and C4-2B cells in an androgen-independent manner.

The presence of androgen caused more extensive apoptosis of LNCaP cells (80% apoptosis) than in the absence of the hormone (50% apoptosis) (Figure 2c). Given the androgen dependence of these cells, the much higher apoptosis of mock-infected cells in the absence of androgen is expected.

### RSV-induced oncolysis of tumors *in vivo *in mice through apoptosis

**Figure 3:**
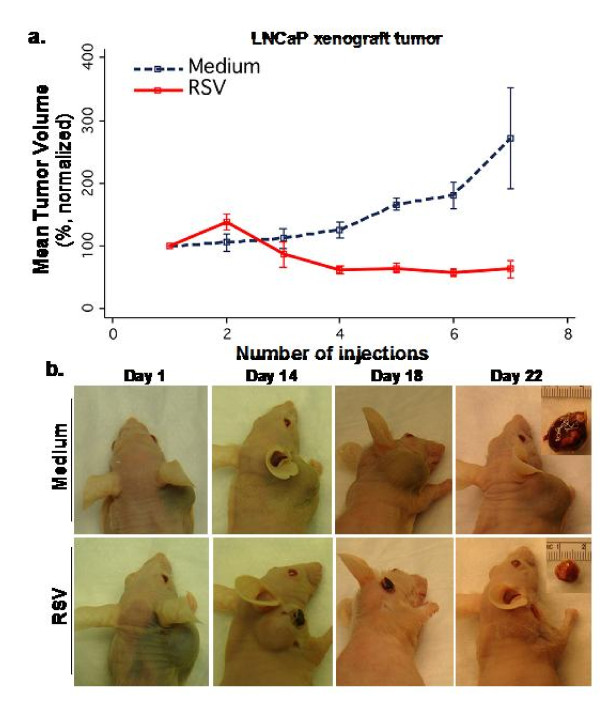
**Androgen-sensitive LNCaP xenograft prostate tumor growth in nude mice following intratumoral injection of RSV**. Tumors were produced by subcutaneous injections of LNCaP cells at sites below the ear. **(a) **Tumor growth kinetics in mice. Intratumoral injections were given at 2 days apart. Tumor volume of each mouse was normalized against its tumor volume at day 1 (starting point) set as 100%. Tumor volumes (normalized) measured on each injection day are shown in the plot. Each treatment group consisted of four representative animals (n = 4) and data represent normalized mean tumor volume trajectories over time. Error bars represent the SE of the mean at each time point. **(b) **Progressively regressed tumor mass after intratumoral injection of RSV. Tumor growth corresponding to injections of RSV or Medium was followed. Day 1 represents the initial injection of RSV or Medium. The Inserts display corresponding tumors extracted at day-22 before sacrifice.

**Figure 4:**
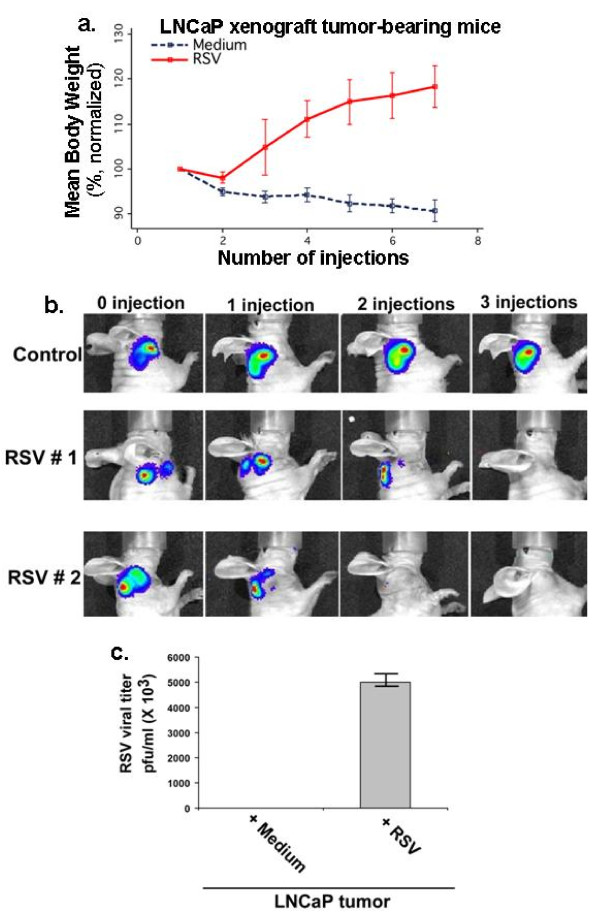
***in vivo *oncolytic activity of RSV against androgen-sensitive xenograft prostate tumor**. **(a) **Body weight of tumor bearing mice measured over 14 day following intratumoral administration of RSV or Medium at 2-day intervals. The body weight data represent the normalized mean body weight trajectories over time. Body weight of each mouse was normalized against its body weight at day 1, which was set as 100%. Error bars represent the SE of the mean at each time point. **(b) **Real-time bioluminescence imaging of xenograft tumors (generated using luciferase expressing LNCaP-Luc-2 cells) in live animal following RSV or medium (control) injections (I.T). Time interval for each injection = 2 days. Whole-body imaging was done with Xenogen IVIS system. **(c) **LNCaP xenograft tumor was injected either with medium (control) or RSV (1 × 10^5 ^pfu) via I.T. After 3 d post-injection, the tumor was surgically removed and RSV titer in the tumor homogenate was determined by plaque assay.

Intratumoral administration of RSV to the subcutaneously produced LNCaP xenograft tumors markedly reduced tumor mass over a three week period, while medium-injected tumors (carrier control) continued to increase in size over time (Figure 3a). Photomicrographic documentation of tumor regression in a representative mouse is shown (Figure 3b). Tumor-bearing nude mice receiving medium injection showed drastic reduction in body weight. This is in contrast to the steady body-weight increase in mice that received RSV into tumors (Figure 4a). We speculate that the decreased body weight (cancer cachexia) is due to the catabolic action of the proteolysis-inducing factor (PIF) on skeletal muscle and possibly, to some extent, also due to the catabolic action of TNF-α (cachectin) on the adipose tissue and skeletal muscle [16]. Thus, the anti-tumor activity of RSV helps relieve tumor-associated pathophysiological ailments.

Real-time bioluminescence imaging in live mice (using Xenogen IVIS imaging system) also showed drastic tumor regression by RSV (Figure 4b). The tumor regression was directly due to lytic viral replication in tumor cells, since high RSV titer was detected in the homogenate of the LNCaP xenograft tumor tissue that was harvested 3 days after a single RSV injection via I.T (Figure 4c).

**Figure 5:**
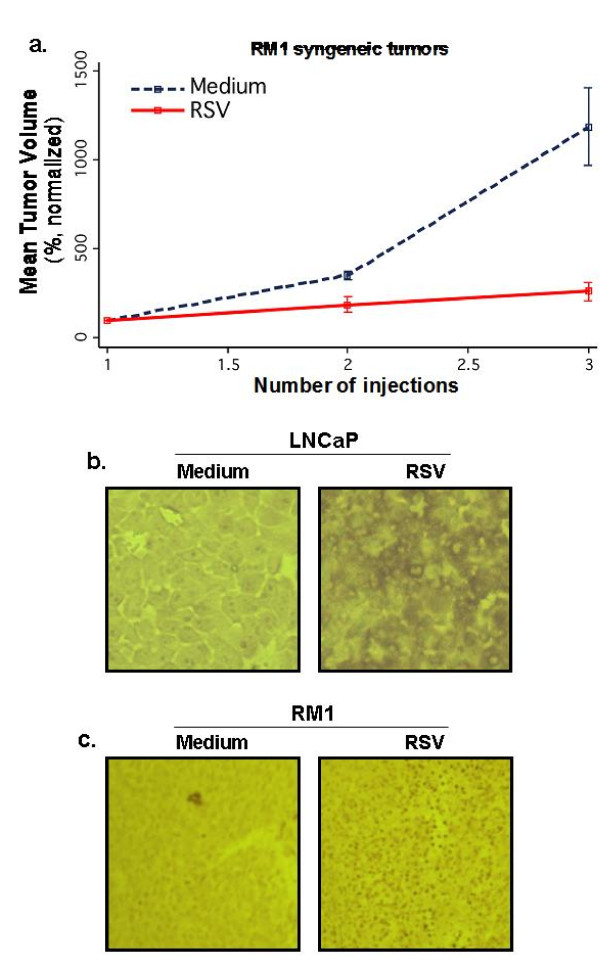
**Growth of prostate tumors in immune-competent C57BL/6J mice and RSV-mediated apoptosis of prostate tumors *in situ***. **(a) **RM1 murine prostate cancer cell tumors, grown subcutaneously in C57BL/6J mice, were injected with RSV or medium (intratumoral). Each treatment group had four representative animals (n = 4) and data represent the normalized mean tumor volume trajectories over time. Tumor volume of each mouse was normalized against its tumor volume at day 1 of injection, which was set as 100%. Error bars represent SE of the mean. **(b, c) **TUNEL assay with prostate tumors derived from LNCaP **(a) **or RM1 **(b) **cells (at 100× magnifications). Tumors, injected with RSV or Medium (control), were excised, processed and tumor sections were analyzed by TUNEL assay. RM1 tumors were derived 12 h after two RSV injections (2 d apart) via intratumoral route. In contrast, LNCaP tumors were derived 12 h after three RSV injections (2 d apart) via intratumoral route.

Furthermore, the oncolytic effect of RSV is not compromised in the immune-competent syngeneic host, since RSV significantly inhibited growth of RM1 murine prostate cancer cell tumors generated in C57BL/6J mice (Figure 5a). We observed that RSV specifically targeted and localized to LNCaP- and RM1-derived prostate tumors since viral gene specific mRNA expression was detected in tumor lysates, but not in lysates of various organs (lungs, kidney, liver, spleen) of infected animals (data not shown). Similar tumor-specific RSV targeting was noted previously in mice harboring PC-3 derived xenograft tumor [7].

Intratumoral RSV injection significantly enhanced apoptosis of tumor cells (shown by *in situ *TUNEL assay) for LNCaP (Figure 5b) and RM1 (Figure 5c) derived tumors compared to control tumors. This concurs with our *in vitro *data of RSV-induced apoptosis in LNCaP and RM1 cells (Figure 1d).

### Sustained tumor remission after RSV administration and subdued immune response to RSV challenge

**Figure 6:**
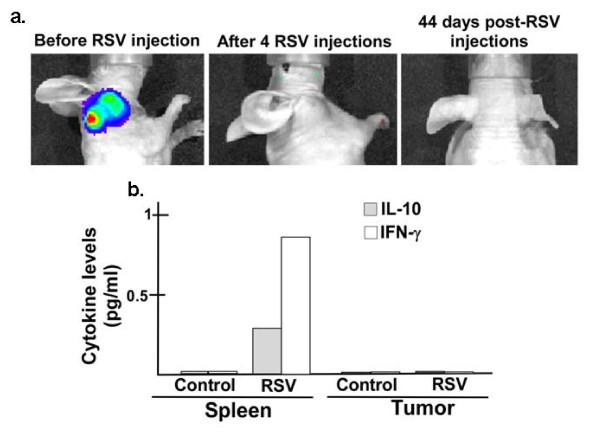
**Sustained tumor remission after RSV administration and subdued immune response to RSV challenge**. **(a) **RSV was administered (via I. T - four injections at 2-day intervals) to xenograft tumor (generated from luciferase expressing LNCaP-Luc2 cells). Following 4 injections, the mouse was observed everyday for a period of 44 days for tumor recurrence. Real-time bioluminescence imaging of xenograft tumors in the live animal was performed with Xenogen IVIS system. **(b) **Levels of interferon-γ (IFNγ) & interleukin-10 (IL-10) in spleen homogenate of normal, C57BL/6J mice (non-tumor bearing) infected with RSV (4 injections of RSV, 2 days apart -10^6 ^pfu/mouse via i.p.) were measured (by ELISA) at the day-14 following last injection. Cytokines were also measured in xenograft prostate tumor (subcutaneous tumor generated using LNCaP cells) 48 hr after RSV injections via I.T. The representative results are from duplicate experiments with similar values.

The therapeutic potential of RSV was further evident from its long-term anti-tumorogenic impact. Xenograft prostate tumors (from luciferase expressing LNCaP-Luc-2 cells), subjected to four RSV injections (at 2 days apart), resulted in tumor regression and the tumor failed to reappear even at 44 days after the final RSV injection (Figure 6a). The long-term tumor remission following RSV treatment underscores the feasibility of developing RSV as an efficient anti-cancer agent.

Immune responses directed against viruses pose a major hurdle in developing efficient oncolyitc viruses with potent anti-cancer property. In that regard, immune response against RSV was minimal, since RSV failed to induce robust immunity following systemic (via intraperitonial or i.p route) infection of normal, immuno-competent C57BL/6J mice that did not host xenograft tumors. Very low levels of Th1 (IFN-γ) and Th2 (IL-10) cytokines (0.25-0.90 pg/ml of IL-10 and IFN-γ respectively, in the spleen homogenate) in infected animals were indicative of weak immune response following systemic RSV infection (Figure 6b). Systemic challenge with various foreign agents (bacteria, virus etc) typically produces 100-3500 pg/ml of IL-10 and IFN-γ in the spleen [17-19].

We also examined whether RSV triggered immune response in the tumor micro-environment. LNCaP xenograft tumors were injected with RSV (two injections via I.T route; 2-day apart) and tumor lysate (collected 2 d after the last injection) was analyzed for the Th1 and Th2 cytokine levels. The almost non-detectable immune mediators (Figure 6b) suggest that tumor regression is not due to host adaptive immunity and self-elimination. These results along with our data showing lytic RSV replication in the tumors (Figure 4c) suggest that tumor regression occurred mostly due to a direct RSV-mediated apoptosis of tumor cells.

### Tumor pathology

**Figure 7:**
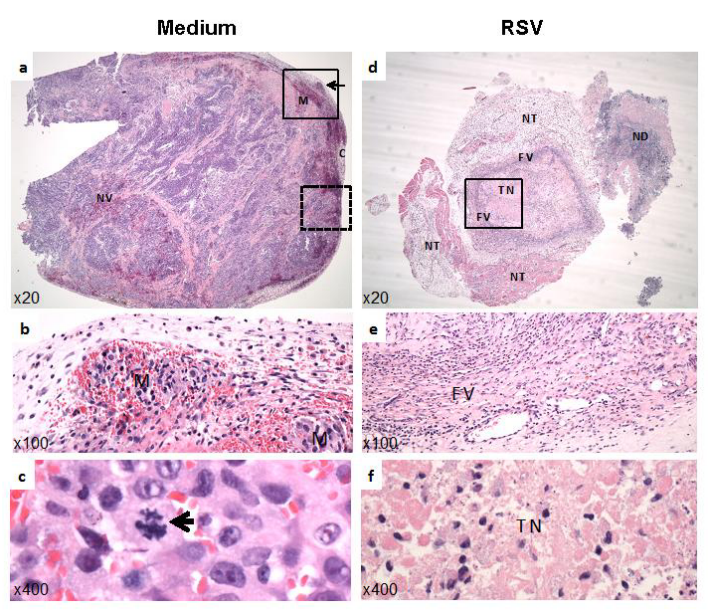
**Histopathology of LNCaP xenograft prostate tumors**. **(a) **Photograph of tumor (at 20× magnification), injected with medium shows an irregular expansible growth and compression (marked as C) of adjacent normal tissue, revascularization (marked as NV) and vascular metastasis (marked as M). **(b) **100× magnification of the upper square in panel-a (solid-line boundary for the demarcated box), showing prominent vascular metastasis (M). **(c) **400× magnification of the lower dashed square (demarcated by the broken-line boundary) in panel-a, showing a typical mitotic figure (see arrow). **(d) **Photograph (at 20X) of RSV-injected tumor. Tumor size is markedly reduced and complete necrosis of the neoplastic cells with minimal fibrovascular encapsulation and pyogranulamatous inflammation is evident. Marked necrosis at the center of the tumor is readily apparent. **(e) **100× magnification and **(f) **400× magnification of the solid-line square in panel-d, highlighting minimal fibrovascular encapsulation, FV (panel-e) and complete tumor necrosis, TN (panel-f). NT: normal tissue. ND: non-tumor debris.

After administering 7 injections of RSV or medium intra-tumorally (injected every other day), tumors were examined for pathological status. The most prominent change for RSV-injected tumors, compared to medium-injected tumors, was complete necrosis with no histologically discernable tumor cells (Figure 7). RSV infected tumors showed markedly reduced size containing a central necrotic core surrounded by a fibrous pyogranulamatous capsule. The normal inflammatory process led to significant healing, as revealed by replacement of the tumor mass by non-malignant tissue (compare Figure 7d with Figure 7a). Histological evaluations of RSV-injected versus medium-injected tumors (3 tumor specimens in each group) are presented in Table 1. Differences between the control and experimental group are easily discernable. Differences were most prominent in the degree of invasiveness, (4, 4 and 5 vs. 0, 0 and 0), mitotic figures per high power field (2, 11 and 22 vs. 0, 0 and 0), necrosis (2, 1 and 2 vs. 5, 5 and 5), compression of adjacent tissue (2, 1 and 2 vs. 0, 0 and 0), mineralization (0, 0 and 0) vs. 4, 3 and 3). Differences for several additional criteria (edema, congestion, hemorrhage, and hemosiderin deposition) were not significant.

**Table 1:** Histological evaluation of Medium- and RSV-injected tumors

Slide ID	Injection	Invasion	m/hpf	Necrosis	Compression	**Min**.	NV
1	Medium	4	2	2	2	0	5
2	Medium	4	11	1	1	0	3
3	Medium	5	22	2	2	0	2
4	RSV	0	0	5	0	4	2
5	RSV	0	0	5	0	3	2
6	RSV	0	0	5	0	3	0

Histological evaluations of RSV-injected versus medium-injected tumors (3 tumor specimens in each group) are presented. Tumor sections were stained with hematoxylin and eosin. For each tumor sample, pathologic changes were graded by a blinded, board certified veterinary pathologist (GBH) as 0 = normal, 1 = minimal, 2 = mild, 3 = moderate, 4 = moderately severe, 5 = severe. Grading criteria were based on invasion of adjacent normal tissue, necrosis, mitotic figures per 20X high power field (m/hpf*), compression of the surrounding tissue, mineralization (min**^§^**) and neovascularization (NV**^II^**).

### Loss of IFN-regulated antiviral defense in LNCaP cells

A critical antiviral role of type-I IFN cytokines in restricting infection from RSV and various other viruses such as measles and vesicular stomatitis virus has been demonstrated [10,20-23]. Therefore, we investigated whether loss of IFN-mediated antiviral defense mechanism would account for differential oncolytic activity in LNCaP versus RWPE-1 cells.

**Figure 8:**
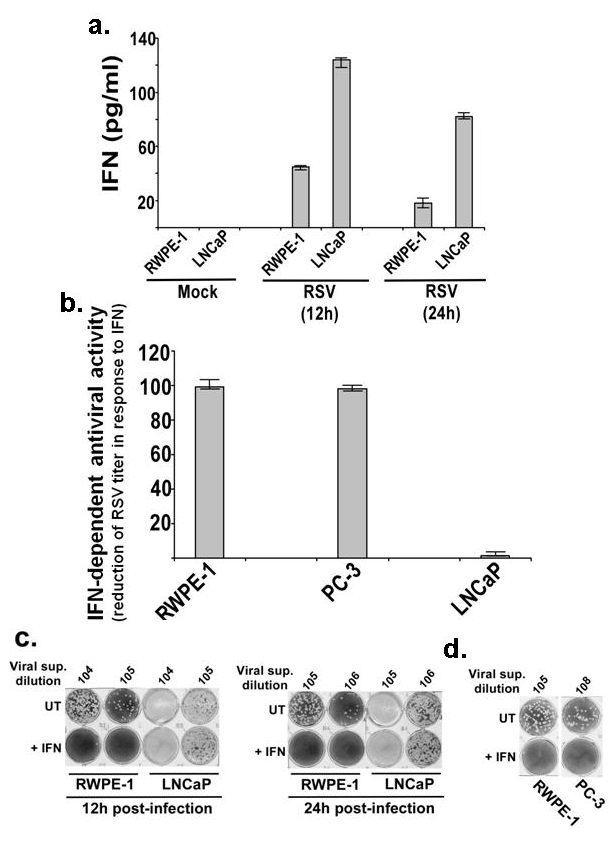
**Interferon response of prostate cancer cells**. **(a) **IFN-β production from mock-infected and RSV-infected RWPE-1 and LNCaP cells was measured at 12 h and 24 h post-infection. Amount of IFN-β deduced from the ELISA assay was expressed as pg/ml and each value represents mean ± standard deviation for three determinations. **(b) **RSV infectivity in untreated (UT) and IFN pre-treated cells was measured by plaque assay at 24 h post-infection. The values are fold reductions in the RSV titer following IFN pre-treatment compared to untreated cells. Mean ± standard deviation for three determinations are shown. **(c) **Plaque assay showing RSV infectivity in untreated (UT) and IFN pre-treated RWPE-1 and LNCaP cells. Culture supernatant collected from RSV infected cells were added to CV-1 cells at various dilutions (1 × 10^4 ^- 1 × 10^6 ^dilutions). **(d) **Plaque assay showing RSV infectivity in untreated (UT) and IFN pre-treated RWPE-1 and PC-3 cells at 24 h post-infection. Culture supernatant collected from RSV infected cells was added to CV-1 cells at various dilutions (1 × 10^5 ^and 1 × 10^8 ^dilutions). For figures (c) and (d) the plaques were observed on methyl-cellulose after crystal-violet staining. Please note that figures (c) and (d) are two separate experiments.

**Figure 9:**
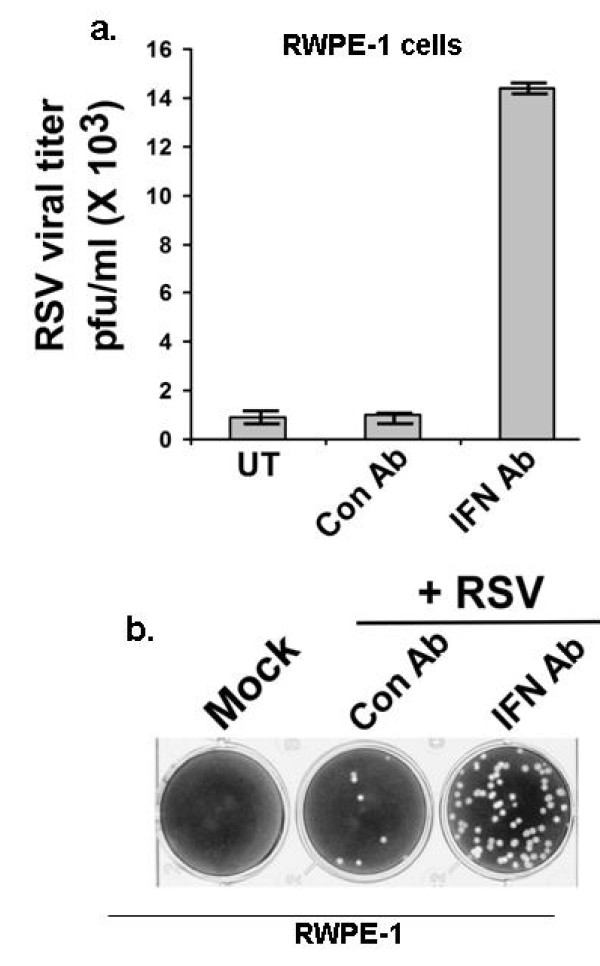
**Interferon response is essential for limiting RSV infection in prostate cells**. **(a) **RSV infection of RWPE-1 in the absence (untreated, UT) or in the presence of either control (Con Ab) or IFN neutralizing (IFN Ab) antibody. Infection was measured by plaque assay at 36 h post-infection. Plaque assay values expressed as pfu/ml represent mean ± standard deviation for three independent determinations. Standard deviations are shown by error bars. **(b) **Plaque assay showing RSV infectivity in Con Ab or IFN Ab treated cells. Culture supernatant from RSV infected RWPE-1 cells (+/- Con Ab or IFN Ab) was added to CV-1 cells at a dilution of 1 × 10^6^. Plaques were observed on methyl-cellulose after crystal-violet staining.

Deregulation of IFN-mediated antiviral response could occur due to either insufficient IFN production from cancer cells, or a dysfunctional IFN-activated JAK/STAT antiviral pathway. RSV-infected LNCaP cells produced high levels of IFN - even more than RWPE-1 cells (Figure 8a). However, the antiviral activity of IFN (as measured from the viral titer of the RSV-infected cells) was at least 100-fold higher in the case of RWPE-1 and PC-3 compared to LNCaP cells (Figure 8b). For these experiments (Figure 8b), cells were pre-treated with IFN for 16 h, followed by RSV infection in the continued presence of IFN. The viral titer was measured by performing plaque assay of medium supernatants. Representative plaque assay shows that IFN treatment caused drastic reduction of RSV infectivity in RWPE-1 (12 h and 24 h post-infection), while failing to significantly inhibit RSV infectivity/replication in LNCaP cells at 12 h and 24 h post-infection (Figure 8c). In contrast to LNCaP cells, PC-3 cells were protected against RSV to the antiviral action of IFN, since IFN treatment of PC-3 cells drastically inhibited virus replication (Figure 8d). In the absence of protection from IFN, RSV has selective growth advantage in LNCaP cells over RWPE-1 cells and PC-3 cells. Indeed, the IFN neutralizing antibody, which inhibited IFN-α/β mediated antiviral activity in RWPE-1 cells, caused significant elevation of the RSV titer in RWPE-1 cells (by approximately 15 fold), representing enhancement of viral infectivity by 750% (Figure 9a). A representative result from plaque assay of RSV-infected RWPE-1 cells that were pretreated with either control antibody or IFN-neutralizing antibody shows elevated viral titer in cells devoid of IFN response (Figure 9b). Results from Figure 9 demonstrate that IFN plays an important role in limiting RSV infection in RWPE-1 and PC-3 cells, whereas lack of this restriction in LNCaP cells associated with excessive viral replication and oncolysis.

**Figure 10:**
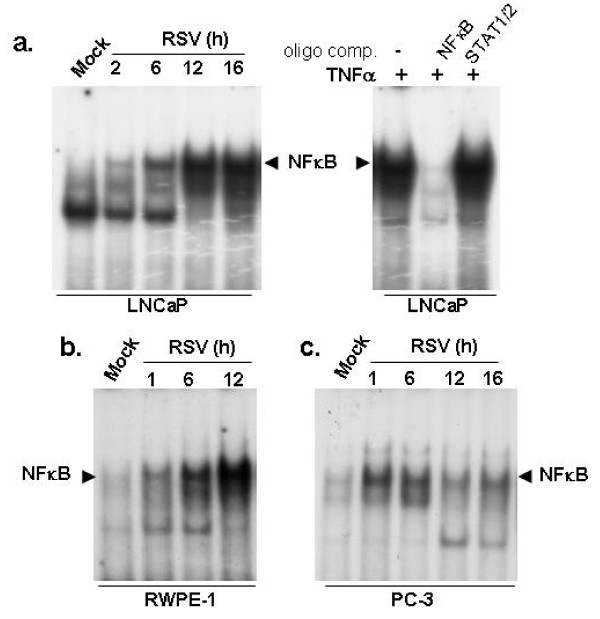
**NF-κB activation in RSV infected prostate cancer cells**. NF-κB specific EMSA of nuclear extracts prepared from mock-infected and RSV-infected LNCaP (**a**) RWPE-1 (**b**) and PC-3 **(c) **cells. Duration of infection (in hours) with RSV is indicated. The NF-κB/DNA EMSA complex is indicated with arrowheads. Competition of the EMSA complex with the cold NF-κB duplex oligonucleotide, but not with cold STAT1/2 oligo duplex in panel (a) ensures the specific nature of the EMSA complex.

We had reported earlier [7] that PC-3 cells are susceptible to RSV-induced apoptosis despite an intact IFN-responsive antiviral response (Figure 8b). This is due to a defect in the NF-κB-mediated antiviral response, which was compromised within 6-12 h post-infection (Figure 10c). However, RSV-infected LNCaP and RWPE-1 cells were not impaired for NF-κB activation (Figures 10a, b).

### Lack of IFN-regulated STAT-1 activation in RSV-infected LNCaP cells

**Figure 11:**
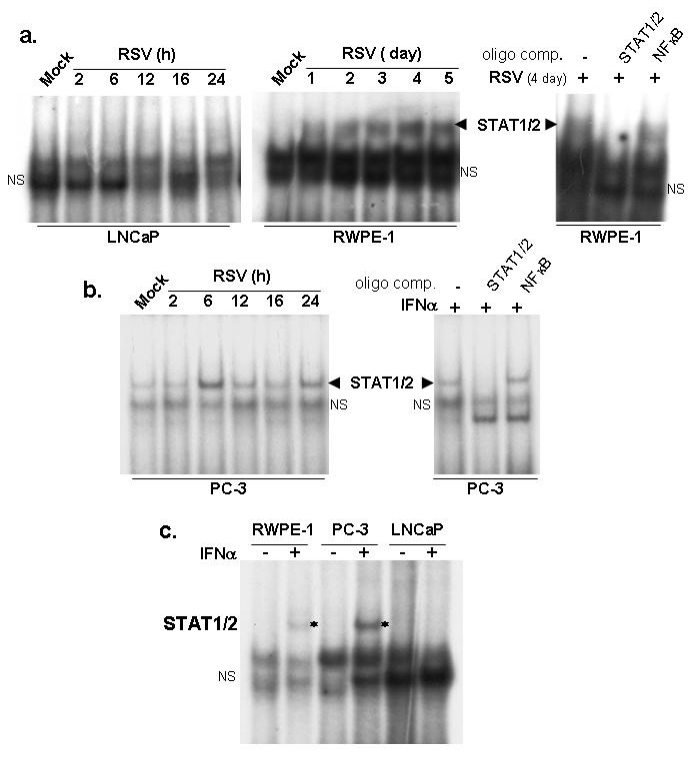
**Impaired IFNα- and RSV-mediated STAT-1 activation in LNCaP cells**. STAT-1-specific EMSA of nuclear extracts prepared from mock-infected and RSV-infected LNCaP and RWPE-1 cells **(a) **PC-3 cells **(b)**. The STAT-1/DNA EMSA complex is indicated with arrowheads. **(c) **STAT-specific EMSA of nuclear extracts prepared from cells treated with (+) or without (-) IFNα for 4 hours. Asterisks represent STAT-1/DNA complex. NS: non specific band.

Following engagement with the cognate IFN-receptor, IFN-α/β stimulates a signaling cascade (JAK/STAT pathway) that culminates in activation of the transcription factors STAT-1 and STAT-2 (STAT-1/2), which translocates to the nucleus to transactivate antiviral genes [24]. Although LNCaP cells secreted high levels of IFN (Figure 8a), unlike RWPE-1 and PC-3 cells, RSV-infected LNCaP cells did not activate STAT-1, revealed from the lack of STAT-1 binding to the cognate DNA element in LNCaP cells (Figure 11a) in contrast to RWPE-1 (Figure 11a) and PC-3 (Figure 11b) cells, which showed robust STAT-1 activation. EMSA complexes in RWPE-1 and PC-3 cells are shown by the arrow, and specificity of the EMSA is shown by the loss of STAT-1-specific EMSA complex in competition assay (Figure 11a, b).

We conclude that inability of LNCaP cells to activate STAT-1 in response to RSV infection is due to a globally dysfunctioning IFN pathway, since IFN treatment did not activate STAT-1 in LNCaP cells (Figure 11c).

## Discussion

Our study has been the first to demonstrate RSV is an oncolytic virus, and this oncolytic activity is functional *in vivo *both in immune-deficient nude mice and in an immune-competent host environment, since RSV inhibited prostate tumor growth in syngeneic C57BL/6J mice. RSV infectivity and the virus-induced apoptotic index *in vitro *were much higher in androgen-dependent LNCaP cells compared to non-tumorigenic RWPE-1 prostate cells. Aberrant type I interferon (IFN)-dependent antiviral defense response [10], which culminated in impaired activation of the STAT-1 transcription factor (STAT-1 is required for expression of IFN-dependent antiviral genes), associated with the high RSV burden in infected LNCaP cells. We conclude that blockade in STAT-1 activation, leading to inhibition in the expression of critical IFN-dependent antiviral genes, accounts for excessive RSV replication leading to apoptosis of LNCaP cells. This is unlike PC-3 androgen-independent prostate cancer cells for which RSV-induced oncolysis was associated with failure in a sustained NF-κB activation, which would cause failure in the induction of NF-κB dependent antiviral genes.

Using IFN-neutralizing antibody, we also provide the first direct evidence (Figure 9) that protection of non-malignant epithelial cells against virus-induced oncolysis is due to IFN-mediated antiviral defense response. Our results further revealed that the oncolytic function of RSV may remain active even when the IFN-regulated antiviral pathway is functional, provided a second defense arm involving NF-κB signaling is deregulated.

Innate immunity is the first line of defense mounted by the host to combat virus infection before an orchestrated adaptive immune response is launched [10,20]. IFN-mediated activation of the JAK/STAT antiviral pathway is recognized as a major antiviral innate immune defense mechanism [24]. In this regard, we have recently demonstrated that RSV-infected lung epithelial cells and immune cells (e.g. macrophages) utilize Nod2 protein as a molecular sensor to induce production of IFN-α/β from infected cells after interacting with viral single-stranded RNA genome and subsequently triggering innate antiviral response [25]. IFN-α/β, which are potent antiviral cytokines produced in infected cells, bind to cognate cell surface receptors on uninfected cells (via autocrine/paracrine action) to induce the JAK/STAT antiviral pathway; which helps promote nuclear translocation and activation of the transcription factors STAT-1 and STAT-2 that in turn activate antiviral genes [24]. We also reported that an IFN-independent innate defense mechanism involving TNF-α -induced activation of NF-κB can restrict virus replication in infected cells due to induction of antiviral genes [15,26]. These two antiviral pathways mediated by IFN (via the JAK/STAT pathway) and TNF-α (via the NF-κB pathway) are activated in infected cells either individually or together to coordinate the transcriptional induction of the antiviral gene network.

A large number of cancer cells are deficient in the IFN signaling cascade [27,28], making many types of cancer cells susceptible to apoptosis by oncolytic viruses. In the context of prostate cancer, our results suggest that both androgen-dependent prostate cancer cells (such as LNCaP cells) and androgen-independent prostate cancer cells (such as PC-3 and RM1 cells) are susceptible to RSV-induced oncolysis. We show that, while IFN production from infected LNCaP cells was normal, IFN failed to activate STAT-1 in LNCaP cells. In fact, it was reported earlier that LNCaP cells fail to express JAK1 [29]. On the other hand, RSV infection and IFN treatment of non-tumorogenic RWPE-1 cells and PC-3 cells was associated with robust STAT-1 activation and protection against RSV-induced apoptosis. We also show that while PC-3 cells respond to IFN and induce DNA-binding activity of STAT-1 (in agreement with previous reports that IFN-treated PC-3 cells are activated for antiviral signaling; [30,31]), impaired NF-κB activation is associated with apoptosis in RSV-infected PC-3 cells. LNCaP cells, on the other hand, were competent to activate NF-κB in response to RSV infection. We speculate that androgen dependence and/or the androgen receptor expression status of prostate cancer cells may influence RSV-mediated modulation of the innate antiviral apparatus (NF-κB activation vs. IFN-mediated JAK/STAT activation). Our results (Figures 8, 9, 10, 11) [7] lead us to conclude that deregulation of the IFN pathway in androgen-sensitive LNCaP prostate cancer cells accounts for loss of STAT-1 activation (and non-expression of antiviral factors), higher RSV replication, induction of apoptosis and reduced cell viability, whereas deregulation of the NF-κB-dependent antiviral defense in androgen-independent PC-3 prostate cancer cells accounts for susceptibility of these cells to RSV-induced apoptosis.

Advanced-stage cancer cells, which continue to express the androgen receptor in a majority of tumor specimens, are resistant to apoptosis from androgen ablation or from the cytotoxicity induced by chemotherapeutic agents. Development of treatment protocols that would promote prostate cancer cell apoptosis and prevent cancer cell progression to androgen independence has remained a major challenge in prostate cancer therapy. To this end, it is tempting to speculate that complete ablation of prostate cancer cells at an early stage, when the cells are still androgen-sensitive, is likely to prevent clonal emergence of androgen-independent prostate cancer cells. The anti-tumor, oncolytic activity of RSV against androgen receptor-negative prostate tumors has additional clinical significance, since reduced or non-detectable androgen receptor expression has been observed in a small percent of metastatic neoplastic foci at distant organ sites from castrate resistant prostate cancer patients [32,33]. The observation that RSV-induced oncolysis of prostate tumors can occur in immune-competent C57BL/6J mice has obvious clinical significance. It is important to mention that systemic delivery of RSV represent a clinically feasible route for therapy. In that regard, we have previously demonstrated that intraperitoneal (i.p.) injections of RSV are effective in causing regression of PC-3 xenograft tumors [7] which are more aggressive than LNCaP xenograft tumors with regard to tumor growth. Thus, studies are underway to examine oncolytic efficacy of RSV against LNCaP tumors following systemic administration. The results from our study suggest that RSV-based therapy has the potential to be a viable strategy for prostate cancer treatment.

## Conclusions

In summary, our study demonstrated the oncolytic activity of RSV promotes selective apoptosis of androgen-sensitive and androgen-refractory prostate cancer cells by exploiting the deficiency in the antiviral signaling propagated by either the IFN-mediated JAK/STAT activation or NF-κB activation in virus infected cells. The host immune response did not interfere with the oncolytic activity of RSV. On the basis of these results we suggest that the oncolytic property of RSV may be useful in the development of virotherapy for noncurative, metastatic prostate cancer.

## Competing interests

The authors declare that they have no competing interests.

## Authors' contributions

All authors have read and approved the final manuscript. The authors made the following contributions to this work. IE responsible for the study design, experimental work, data evaluation and analysis and helped write the manuscript. TH was involved in experimental design, experimental work and critique. AS performed the interferon assays and viral titer assays. IB was involved in the animal experiments. YK and GBH performed the histology analysis. BC and SB were the research supervisors and participated in the study design, assessment of the results, and writing the manuscript.

## Pre-publication history

The pre-publication history for this paper can be accessed here:

http://www.biomedcentral.com/1471-2407/11/43/prepub
